# Effects of reductive soil disinfestation on the composition of the soil microbial community in degraded facility cucumber

**DOI:** 10.1128/spectrum.00395-25

**Published:** 2026-01-26

**Authors:** Botong Liao, Guoping Hu, Zhihua Liu, Ximao Yu, Mingying Wen, Yanghua Liu, Lin Chen

**Affiliations:** 1 Hengyang Vegetable Research Institution of Hunan Province, Hengyang, China; Università degli Studi di Napoli Federico II, Naples, Italy

**Keywords:** reductive soil disinfestation, organic materials, microbial community, vegetable straw, cucumber

## Abstract

Reductive soil disinfestation (RSD) is an environmentally friendly method that can alleviate continuous cropping obstacles. Organic material application is crucial for ensuring RSD success. However, whether RSD treatment involving vegetable straw as organic material can alleviate continuous cropping obstacles in greenhouse cucumber remains unclear. Herein, we added 25 t∙ha^−1^ of tomato, bitter melon, and pepper straw to soil as organic material for RSD treatment of greenhouse soil, respectively. The results revealed that RSD treatment increased soil pH; organic matter, available nitrogen, and available potassium contents; and sucrase, urease, and catalase activities. Principal coordinate analyses revealed that the bacterial and fungal community compositions were greatly altered and that the fungal and bacterial Chao1 and Shannon indices decreased. The relative abundance of beneficial microorganisms such as *Bacillus*, *Arthrobacter*, *Streptomyces*, and *Penicillium* increased, whereas that of harmful microorganisms such as *Fusarium* decreased. RSD treatment promoted cucumber growth and development and significantly increased yield. On the basis of the RSD treatment effects on soil properties and cucumber growth and development, using pepper straw as organic material for RSD treatment was the most effective. A year of field trials revealed that adding vegetable straw as organic material for RSD treatment improved chemical properties and enzyme activities, altered the cucumber soil microbial community, and increased the cucumber yield. However, the long-term effects of RSD treatment on soil properties and microbial communities must be studied further.

**IMPORTANCE:**

Driven by both economic benefits and market demand, intensive facility agriculture, characterized by uninterrupted monocultures, has become a significant aspect of contemporary agricultural practices. Reductive soil disinfestation (RSD) is an effective agricultural practice, and the application of organic material is crucial for achieving RSD success. Our research revealed that the use of vegetable straw as organic material for RSD treatment can effectively alleviate continuous cropping obstacles in greenhouse cucumber cultivation.

## INTRODUCTION

Cucumber is an important vegetable crop that is widely grown in China, which exhibits the largest planting area and the broadest range of cultivation (1). The total cucumber production in China in 2018 reached approximately 56 million tons within an area of approximately 1 Mha (2). Driven by economic interests and the scarcity of land resources, monoculture continuous cropping is the main cucumber cultivation method (3, 4), whereas long-term monoculture continuous cropping decreases crop yield and quality; this phenomenon is referred to as continuous cropping obstacles (5, 6). Research has revealed that changes in soil microbial communities, chemical properties, and enzyme activity are important factors associated with the occurrence of continuous cropping obstacles (7–9); for example, soil acidification is the main factor correlated with garlic continuous cropping obstacles, and the activities of catalase and urease (UE) are lower in cropping obstacle-affected soil than in healthy control soil (10). In soil continuously cultivated with pepper, the abundance levels of *Fusarium* and *Pseudallescheria* in soil increase, and *Fusarium* is a pathogen that causes crop diseases (11).

Reductive soil disinfestation (RSD) is also referred to as biological soil disinfestation or anaerobic soil disinfestation. In the 21st century, RSD was developed in the Netherlands (12) and Japan (13). RSD is a preplanting soil treatment method that involves adding easily degradable organic materials to the soil, saturating the soil with water, and sealing the treated soil with a plastic film to create a strongly reducing and anaerobic environment (14). Most soil-borne pathogens are aerobic, and the anaerobic environment created by RSD treatment is lethal to some soil pathogens, can alter soil microbial communities, and can affect soil chemical properties and soil enzyme activity (15). Research has shown that RSD treatment can effectively alleviate the continuous cropping obstacles associated with tobacco (16) and lisianthus (17). In general, liquid and easily degradable compounds, such as diluted ethanol and molasses, and solid agricultural wastes, such as livestock excrement and plant residues, are the main organic materials for RSD treatment (18, 19). The carbon-to-nitrogen ratio (C/N ratio) in organic materials is an important factor, and organic materials with low C/N ratios are more effective for RSD treatment (20, 21).

Vegetable straw is agricultural waste produced in the vegetable production process; according to statistics, the annual production of vegetable straw in China exceeds 360 million tons, with more than 60% of the produced waste abandoned without treatment, resulting in an enormous waste of agricultural resources (22). Moreover, vegetable straw is easily accessible and exhibits a low C/N ratio (23). Vegetable straw contains carbon (40%–50%), nitrogen (0.6%–1.0%), phosphorus (0.45%–2.0%), potassium (14%–25%), and micronutrients, which are essential for crop growth (24). Studies have demonstrated that the incorporation of vegetable straw can increase the soil buffering capacity, increase the contents of soil organic matter and available nutrients, increase the diversity of soil microorganisms, and improve community structure. In addition, the incorporation of vegetable straw can significantly increase crop root vigor and increase yield (23). We hypothesize that using vegetable straw as an organic material for RSD treatment can alleviate the continuous cropping obstacles associated with cucumbers in greenhouses. This study aims to (i) investigate the changes in soil properties and microbial communities in cucumber soil after RSD treatment; (ii) evaluate the changes in cucumber growth, development, and yield after RSD treatment; and (iii) explore the relationships among soil properties, microbial communities, and cucumber yield. We hope to provide a new technology to alleviate the continuous cropping obstacles of cucumbers in greenhouses.

## MATERIALS AND METHODS

### Study area and vegetable straw preparation

This study was conducted at the Hengyang Vegetable Research Institute (112°32′92″ E, 26°9245″ N), which is located in Hengyang City, Hunan Province, China. This area receives an average annual rainfall of 1,133.8 mm and exhibits a temperature of 18.2°C, with 1,663.5 h of full sunlight per year and a 293-day frost-free period. The soil texture is sandy loam (61.3% sand, 30.3% silt, and 8.4% clay). Analysis of the soil chemical properties determined the pH to be 5.82, and the soil organic matter (SOM), available nitrogen (AN), available phosphorus (AP), and available potassium (AK) contents were 31.12 g∙kg^−1^, 164.68 mg∙kg^−1^, 187.31 mg∙kg^−1^, and 179.71 mg∙kg^−1^, respectively.

Tomato straw (TS), bitter melon straw (BS), and pepper straw (PS) were obtained from Hengyang Vegetable Research Institute, and their carbon and nitrogen contents were determined (on a dry basis, Table 1). The vegetable straw was then crushed (particle size <5 mm).

**TABLE 1:** Carbon and nitrogen contents of three vegetable straws^*a*^

Treatment	Total carbon (mg∙g)	Total nitrogen (mg∙g)	C/N
TS	444.82 ± 23.46 b	19.88 ± 1.58 b	22.38 ± 2.51 b
BS	526.00 ± 30.92 a	34.57 ± 2.12 a	15.22 ± 1.79 c
PS	498.87 ± 27.86 ab	17.91 ± 1.90 b	27.85 ± 2.34 a

^*a*^ Different letters within a column indicate significant differences at *P* < 0.05, as determined by Duncan’s test, and all the data points are presented as the mean ± standard deviation (*n* = 3).

### Experimental design

The experiments were conducted on 10 July 2023 in a greenhouse at the Hengyang Vegetable Research Institute. Six consecutive cucumber crops were planted in this greenhouse, and severe continuous cropping obstacles occurred, manifested as slow growth and decreased cucumber yields. Fifteen plots were included in the experiment, and each plot was 5.5 m^2^ (5 m length × 1.1 m width). The experiment involved a randomized complete block design with three replicates, and the following five treatments were established: (i) untreated soil (CK); (ii) flooded and mulched soil with plastic film (CKF); (iii) soil supplemented with 25 t∙ha^−1^ of tomato straw, flooded, and mulched with plastic film (TS); (iv) soil supplemented with 25 t∙ha^−1^ of bitter melon straw, flooded, and mulched with plastic film (BS); and (v) soil supplemented with 25 t∙ha^−1^ of pepper straw, flooded, and mulched with plastic film (PS). On 31 July 2023, the plastic film was removed to allow the soil to dry naturally. On 20 August 2023, soil samples were collected from 10 randomly selected points in each plot with a stainless steel sampler (diameter 2.5 cm) at a depth of 0–20 cm and mixed into composite samples. The collected soil samples were sieved (2 mm mesh) and divided into two subsamples: notably, one subsample was air-dried to determine its soil chemical properties and soil enzyme activities, and the other subsample was stored at −80°C until DNA analysis.

On 2 September 2023, 30-day-old cucumber seedlings were transplanted into each plot with a spacing of 60 × 60 cm, with 16 plants of the variety Jiwang cucumber planted in each plot. During vegetable cultivation, a compound fertilizer (N:P:K = 16:16:16) was applied two times per month, with an annual application rate of approximately 2 t∙ha^−1^. Cucumbers were harvested from 7 October to 2 November 2023. On 2 November 2023, growth indices (stem diameter, plant height, and total biomass per plant) and the yield of the cucumber plants were measured, and the cucumber rhizosphere soil was sampled and stored at 4°C and −80°C for subsequent analysis. After cucumber planting, the plants in the CK, CKF, TS, BS, and PS treatments were labeled as qCK, qCKF, qTS, qBS, and qPS treatments, respectively.

### Analysis of plant growth and soil chemical properties

The height of the cucumber plants was measured using a tape measure. The stem diameter of the cucumber plants was measured using a Vernier caliper. The total biomass per plant and yield of the cucumber plants were determined using a tray balance. Soil pH was measured with a calibrated Metro-pH320 pH meter (Mettler-Toledo Instruments, Ltd., Shanghai, China) at a soil:water ratio of 1.0:2.5 (25). The soil SOM content was quantified through the titration method using acidified potassium dichromate (K_2_Cr_2_O_7_-H_2_SO_4_). The AN content in the soil was determined via the alkaline hydrolysis diffusion method. The AP content in the soil was extracted using 0.5 M NaHCO_3_ and quantified using the Olsen-P method, with spectrophotometric analysis conducted at 880 nm. The AK content in the soil was extracted using 1 M NH_4_OAc (pH = 7.0). The AK content was determined photometrically with a Fame spectrophotometer. The detailed experimental procedures for soil nutrient analysis were based on those used for soil agro-chemistry analysis (26).

### Soil enzyme activity analysis

Sucrase (SC) was quantified via the colorimetric method using 3,5-dinitrosalicylic acid, with activity defined as the production of 1 mg of reducing sugars per gram of soil per day at 37°C. UE was quantified on the basis of indophenol blue colorimetry, with activity defined as the production of 1 µg of NH_3_-N per gram of soil per day. Acid phosphatase (ACP) activity was quantified via the disodium phenyl phosphate colorimetry method, with enzyme activity defined as the release of 1 nmol of phenol per gram of soil per day at 37°C. The activity of catalase (CAT) was quantified via colorimetric analysis, with activity defined as the catalytic degradation of 1 µmol of H_2_O_2_ per gram of air-dried soil sample per day (27, 28).

### Soil microbial DNA extraction and sequencing

Total soil DNA was extracted using an EZNA soil DNA kit (Omega Bio-Tek, Norcross, GA, USA). The DNA extract was analyzed on a 1% agarose gel to assess its concentration and purity, which were subsequently determined using a NanoDrop 2000 UV–Vis spectrophotometer (Thermo Scientific, Wilmington, SA, USA). Polymerase chain reaction (PCR) amplification of the 16S rRNA gene was performed using 338F (5′-ACTCCTACGGGAGGCAGCAG-3′) and 806R (5′-GGACTACHVGGGTWTCTAAT-3′) in the bacteria. PCR amplification of the ITS gene was performed using ITS1F (5′-CTTGGTCATTTAGAGGAAGTAA-3′) and ITS2R (5′-GCTGCGTTCTTCATCGATGC-3′) in the fungi. Bacterial 16S rRNA and fungal ITS genes were analyzed as described by Zhan et al. (29) and Tan et al. (21). The amplified products were subsequently sequenced on the Illumina MiSeq PE300 platform (Illumina, San Diego, CA, USA). FLASH (v.1.2.11) was employed to merge the raw sequences generated from MiSeq paired-end sequencing. UPARSE software (v.11) was used to merge and classify the obtained sequences into operational taxonomic units (OTUs) on the basis of 97% similarity (30). Bacteria and fungi were compared with the Silva and Unite databases, respectively, with confidence thresholds of 70% (31).

### Statistical analysis

Excel 2019 and IBM SPSS Statistics 22 were used to analyze the experimental data. The normality and homoscedasticity of the data were determined by Kolmogorov‒Smirnov and Levene’s tests, respectively, with SPSS software. Duncan’s test and independent sample *t*-tests were used to analyze whether significant differences existed between different treatments. Principal coordinate analysis (PCoA) based on the Bray–Curtis distance was conducted to examine the differences in bacterial and fungal community compositions among the treatments. PCoA was performed using R software (v.4.2.2); the fungal and bacterial richness was estimated using Chao1 indices; and diversity was estimated using the Shannon index (32). The relationships between the measured variables were analyzed using Pearson’s correlation coefficient analysis (33). Venn diagrams and microbial abundance maps were created using Origin Pro 2022 (34).

On the basis of the results of Spearman’s correlation analysis, cooccurrence networks of bacteria and fungi were constructed at the OTU level. OTUs with a relative abundance sum of <0.01% were removed, and networks of significant correlations (|*r*| > 0.8, *P* < 0.01) and several network topological parameters were visualized and calculated on the Gephi platform (v.0.10.1) (35). The bacterial community functions were predicted using PICRUSt2 software in combination with the Kyoto Encyclopedia of Genes and Genomes (KEGG) database (36). Fungal community function was predicted using the FUNGuild database (37).

## RESULTS

### Soil chemical property analysis

The different treatments significantly affected the soil chemical properties (Table 2). Compared with those in the CK treatment, the soil pH values and SOM, AN, and AK contents significantly increased (*P* < 0.05) in the TS, BS, and PS treatments, whereas the soil AP content significantly decreased (*P* < 0.05). The pH, SOM, AN, and AK levels of the soil were the highest in the PS treatment. Compared with those in the TS and BS treatments, the soil pH, AN, and AK levels in the PS treatment were significantly greater (*P* < 0.05). The AP content in soil in the qTS, qBS, and qPS treatments was significantly lower (*P* < 0.05) than that in the qCK treatment. The soil SOM content in the qPS-treated soil was significantly greater (*P* < 0.05) than those in the qTS- and qBS-treated soils, whereas no significant difference was observed between soil samples from the qTS and qBS treatments.

**TABLE 2:** Chemical properties of the soils after soil treatment and after pepper planting^*a*^^,*b*^

	Treatment	pH	SOM (g∙kg^−1^)	AN (mg∙kg^−1^)	AP (mg∙kg^−1^)	AK (mg∙kg^−1^)
After soil treatment	CK	5.82 ± 0.09 d	31.12 ± 1.23 d	164.68 ± 4.09 e	187.31 ± 6.28 a	179.71 ± 3.14 e
	CKF	6.21 ± 0.06 c	33.46 ± 1.32 c	174.51 ± 2.14 d	175.02 ± 6.03 b	201.79 ± 5.48 d
	TS	6.56 ± 0.05 b	37.43 ± 1.06 ab	206.15 ± 3.55 b	172.84 ± 4.80 bc	244.31 ± 7.03 b
	BS	6.46 ± 0.04 b	36.32 ± 1.14 b	190.86 ± 3.91 c	162.72 ± 4.89 c	231.10 ± 8.66 c
	PS	6.86 ± 0.05 a	39.18 ± 1.35 a	216.2 ± 3.45 a	169.48 ± 6.15 bc	254.91 ± 6.27 a
After cucumber planting	qCK	5.46 ± 0.05 e	34.51 ± 1.61 c	195.68 ± 5.25 d	210.89 ± 5.70 a	226.81 ± 6.45 e
	qCKF	5.67 ± 0.06 d	35.16 ± 1.81 c	208.04 ± 4.33 c	203.99 ± 4.56 b	236.61 ± 5.54 de
	qTS	6.39 ± 0.04 b	39.11 ± 1.05 b	222.29 ± 3.64 b	188.51 ± 8.88 c	257.64 ± 7.79 b
	qBS	6.21 ± 0.10 c	38.16 ± 1.12 b	218.56 ± 5.32 b	190.21 ± 8.87 c	243.24 ± 5.44 c
	qPS	6.68 ± 0.06 a	44.14 ± 1.98 a	232.17 ± 4.20 a	172.46 ± 5.30 d	269.93 ± 3.52 a

^*a*^ Different letters within a column indicate significant differences at *P* < 0.05, as determined by Duncan’s test, and all the data points are presented as the mean ± standard deviation (*n* = 3).

^*b*^ AK, available potassium; AN, available nitrogen; AP, available phosphorus; SOM, soil organic matter.

### Soil enzyme activity

Significant differences (*P* < 0.05) in the activity of soil SC, UE, ACP, and CAT were found under the different treatments (Table 3). Specifically, compared with those in the CK treatment, the soil SC, UE, and CAT activities in the other treatments significantly increased (*P* < 0.05). The SC, UE, and CAT activities of the soil were highest in the PS treatment, compared with those in the PS treatment; the soil SC activity significantly decreased (*P* < 0.05) by 8.37% and 9.26% in the TS and BS treatments, respectively, but the difference between the TS and BS treatments was not significant. Compared with those in the qCK treatment, the soil SC and UE activities in the qTS, qBS, and qPS treatments significantly increased (*P* < 0.05), whereas the ACP activity significantly decreased (*P* < 0.05). Compared with that in the qPS treatment, the soil CAT activity in the qBS treatment significantly decreased (*P* < 0.05) by 6.39%, and the soil CAT activity in the qPS treatment did not significantly differ but was greater than that in the qTS treatment.

**TABLE 3:** Soil enzyme activity of the soils after soil treatment and after pepper planting^*a*^^,*b*^

	Treatment	SC (U∙g^−1^)	UE (U∙g^−1^)	ACP (nmol∙d∙g^−1^)	CAT (μmol∙d∙g^−1^)
After soil treatment	CK	25.56 ± 0.72 d	2,418.49 ± 80.86 e	39,478.62 ± 746.04 a	32.71 ± 0.45 e
	CKF	27.64 ± 0.84 c	2,654.29 ± 53.99 d	36,460.00 ± 1,072.37 bc	37.83 ± 0.61 d
	TS	30.66 ± 0.75 b	3,242.23 ± 72.75 b	36,024.72 ± 901.18 c	45.50 ± 0.58 b
	BS	30.36 ± 0.67 b	3,043.06 ± 88.16 c	38,240.18 ± 1,388.57 ab	38.92 ± 0.20 c
	PS	33.46 ± 0.77 a	3,411.02 ± 84.41 a	21,724.59 ± 1,168.03 d	46.83 ± 0.43 a
After cucumber planting	qCK	22.63 ± 0.92 d	2,990.73 ± 56.78 c	35,937.60 ± 1,277.11 a	45.24 ± 1.41 c
	qCKF	24.68 ± 0.62 c	3,092.97 ± 75.82 c	29,401.17 ± 1,186.81 b	45.30 ± 0.88 c
	qTS	27.05 ± 0.57 b	3,318.99 ± 67.56 b	28,401.97 ± 1,308.31 bc	48.20 ± 0.52 ab
	qBS	26.02 ± 0.75 bc	3,251.55 ± 60.41 b	26,208.32 ± 1,196.99 cd	46.70 ± 1.52 bc
	qPS	29.39 ± 0.79 a	3,531.60 ± 74.35 a	25,736.01 ± 1,388.67 d	49.89 ± 0.98 a

^*a*^ Different letters within a column indicate significant differences at *P* < 0.05, as determined by Duncan’s test, and all the data points are presented as the mean ± standard deviation (*n* = 3).

^*b*^ ACP, acid phosphatase; CAT, catalase; SC, sucrase; UE, urease.

### Microbial diversity analysis

#### Comparison of OTUs

**Fig 1:**
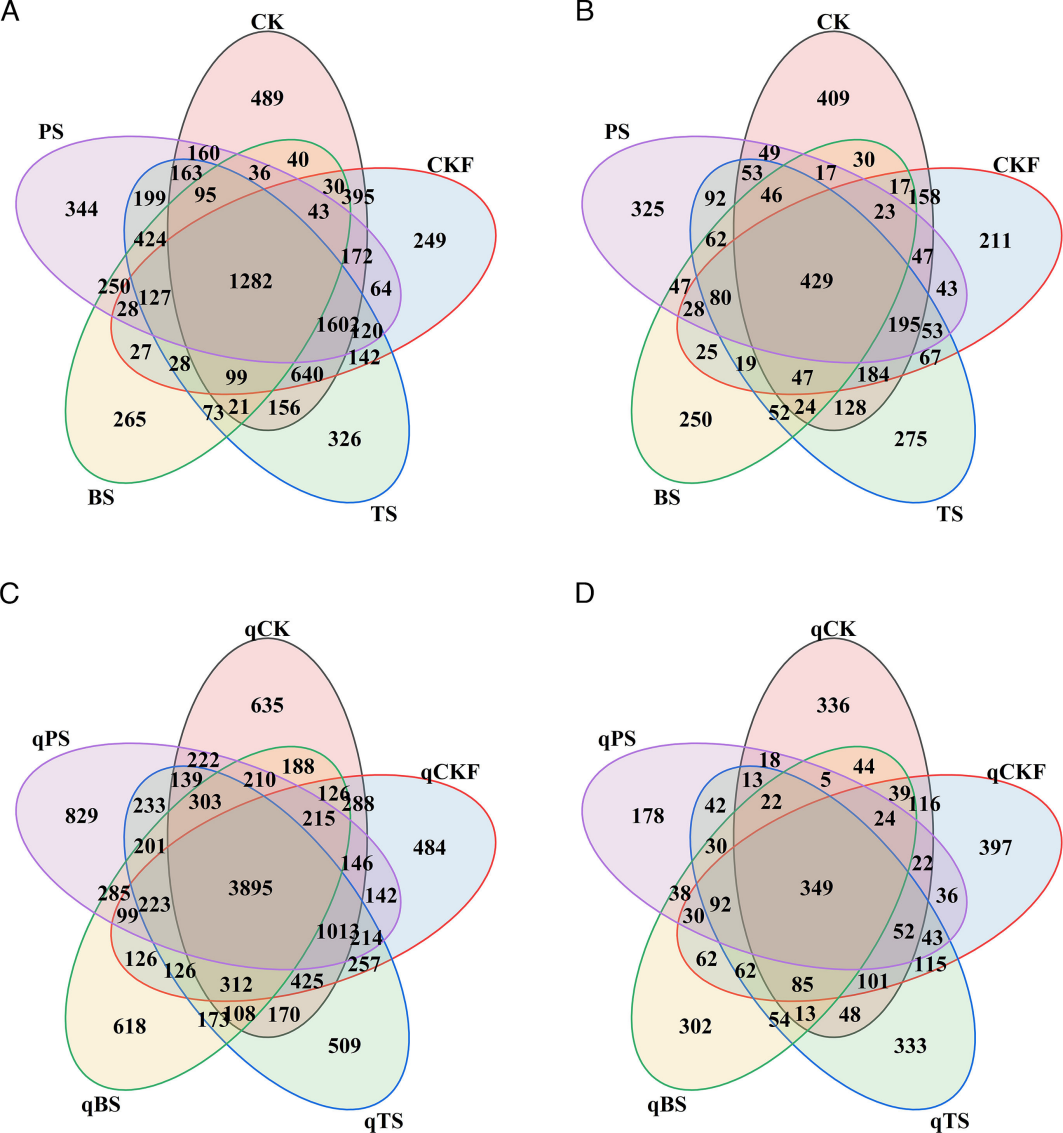
Venn diagrams of bacteria and fungi in different soils after soil treatment (**A and B**) and after cucumber planting (**C and D**).

After the soil treatment, we obtained 1,792,121 high-quality bacterial 16S rRNA gene sequences and 2,315,733 high-quality fungal ITS sequences from 15 soil samples. A total of 8,089 bacterial and 3,485 fungal OTUs with 97% similarity were acquired. After cucumber planting, we obtained 3,048,035 high-quality bacterial 16S rRNA gene sequences and 2,540,207 high-quality fungal ITS sequences from 15 soil samples. A total of 12,915 bacterial and 2,107 fungal OTUs with 97% similarity were acquired. The distribution characteristics of OTUs between the different treatments were visualized in a Venn diagram (Fig. 1).

### Alpha diversity

As indicated in Table 4, the coverage index of each sample was greater than 99%, indicating that the sequencing depth covered most of the samples and could truly and effectively reflect the microbial community composition of the soil samples. In this study, the Chao1 and Shannon indices were chosen to analyze the alpha diversity of the soil microorganisms, with higher Chao1 values indicating greater community richness and higher Shannon values indicating greater community diversity. Compared with those in the CK treatment, the bacterial Chao1 and Shannon index values significantly decreased (*P* < 0.05) in the BS and PS treatments, and the BS treatment resulted in the lowest Chao1 and Shannon index values for bacteria, which were significantly lower than those in the PS treatment. Among the different treatments, no significant differences in the Shannon index of the fungi were observed.

**TABLE 4:** Richness and diversity indices of bacteria and fungi in the soils after soil treatment and after pepper planting^*a*^

	Treatment	Bacteria			Fungi		
		Chao1	Shannon	Coverage (%)	Chao1	Shannon	Coverage (%)
After soil treatment	CK	4,804.22 ± 109.34 a	9.64 ± 0.05 a	99.01	1,192.96 ± 275.33 a	6.42 ± 0.50 a	99.86
	CKF	4,366.73 ± 80.59 b	9.39 ± 0.06 ab	99.09	1,030.74 ± 178.68 a	5.36 ± 0.50 a	99.88
	TS	4,463.46 ± 152.25 ab	9.43 ± 0.22 ab	99.09	1,123.55 ± 68.42 a	6.30 ± 0.17 a	99.86
	BS	2,325.45 ± 138.30 c	7.40 ± 0.39 c	99.51	690.11 ± 136.50 b	5.35 ± 1.04 a	99.94
	PS	4,186.54 ± 193.16 b	8.83 ± 0.58 b	99.11	951.69 ± 170.97 ab	5.88 ± 0.89 a	99.90
After cucumber planting	qCK	6,577.17 ± 166.55 a	10.21 ± 0.03 ab	99.43	798.84 ± 254.19 ab	6.90 ± 0.72 a	99.95
	qCKF	6,375.22 ± 93.23 a	10.25 ± 0.21 a	99.37	955.11 ± 127.98 a	6.82 ± 0.54 a	99.93
	qTS	6,302.95 ± 297.18 a	10.15 ± 0.06 ab	99.34	679.27 ± 251.04 ab	6.77 ± 0.09 a	99.95
	qBS	5,163.36 ± 134.56 b	10.19 ± 0.09 ab	99.57	660.10 ± 106.87 ab	6.04 ± 1.33 a	99.95
	qPS	6,095.42 ± 238.35 a	9.81 ± 0.41 b	99.51	552.79 ± 82.95 b	6.33 ± 0.56 a	99.96

^*a*^ Different letters within a column indicate significant differences at *P* < 0.05, as determined by Duncan’s test, and all the data points are presented as the mean ± standard deviation (*n* = 3).

### Dissimilarity of the soil microbial community

**Fig 2:**
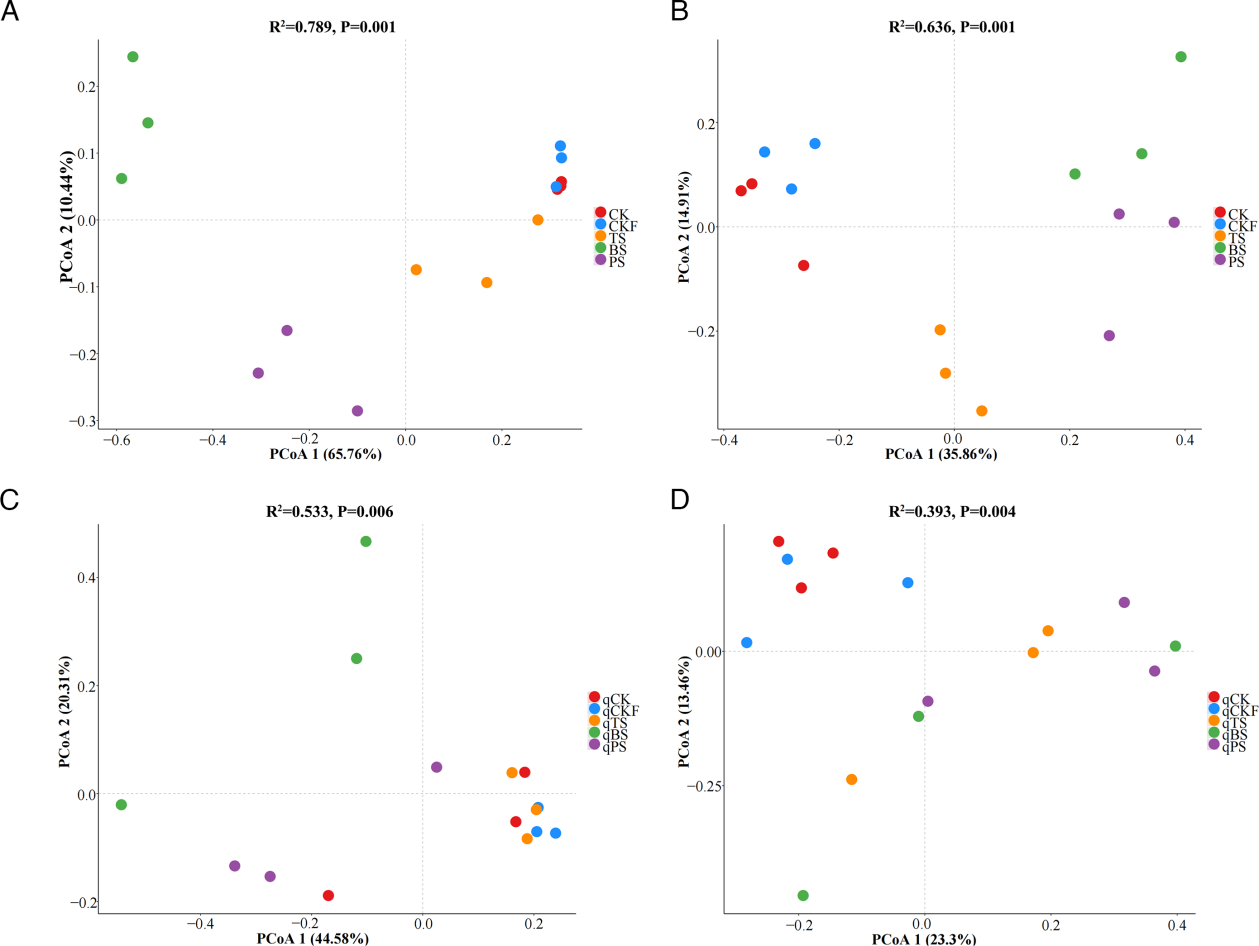
Principal coordinate analysis (PCoA) of soil bacterial and fungal communities on the basis of the Bray–Curtis distance after soil treatment (**A and B**) and cucumber planting (**C and D**).

As shown in Fig. 2, the diversity of bacterial and fungal communities across the different soil treatments was assessed via PCoA. Compared with those in the CK treatment, the bacterial and fungal communities in the BS and PS treatments significantly differed, but no significant differences were found between the CK and CKF treatments (Fig. 2A and B). The differences in soil bacterial diversity among the qCK, qCKF, qTS, and qPS treatments were not significant, whereas significant differences in bacterial diversity were observed between the qBS and qCK treatments (Fig. 2C). Compared with those in the qCK and qCKF treatments, the fungal communities in the qTS, qBS, and qPS treatments significantly differed (Fig. 2D).

### Microbial community composition analysis

#### Analysis of microbial community composition at the phylum level

**Fig 3:**
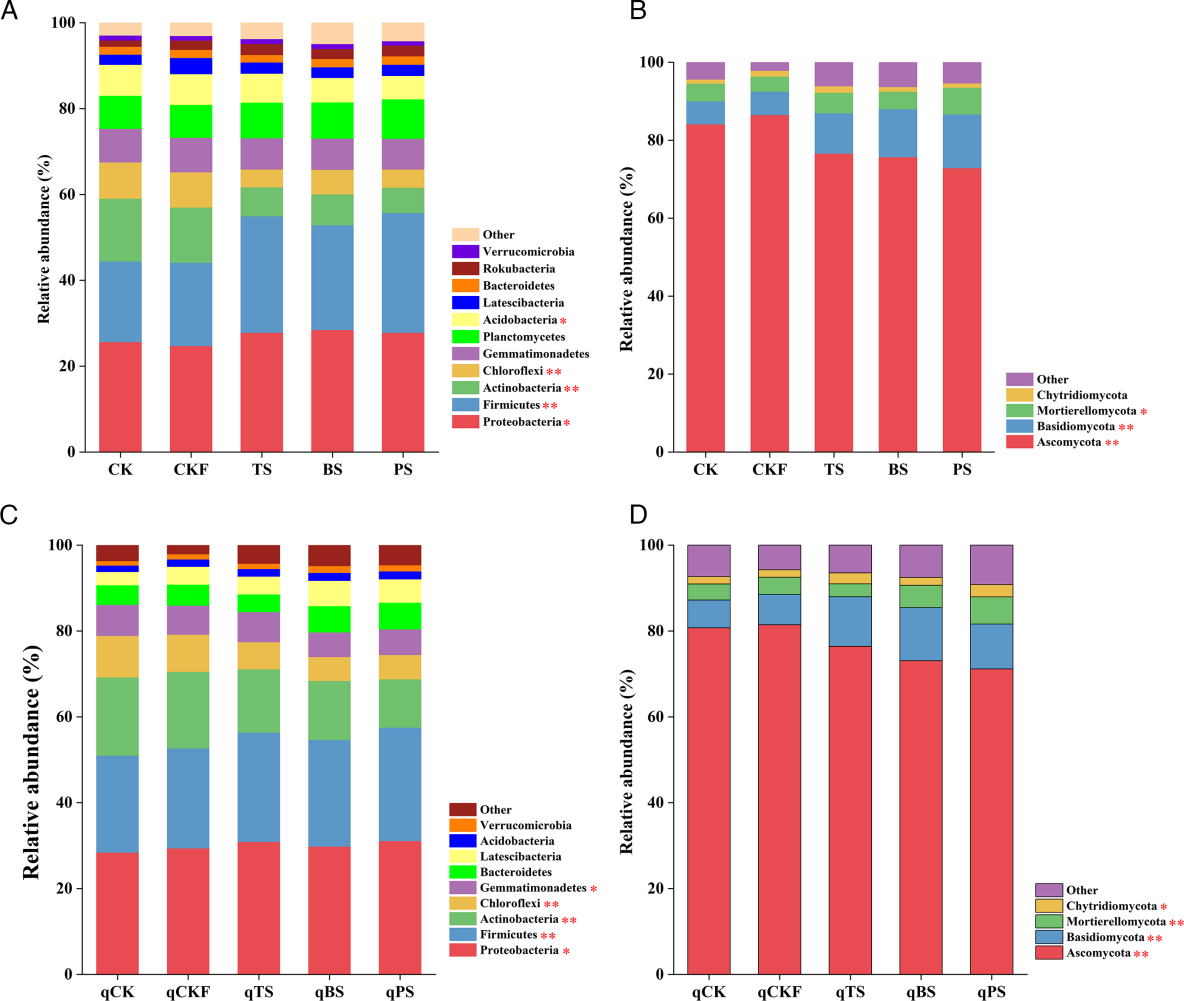
Relative abundance (%) of the bacterial and fungal phyla in the soils under different treatments after soil treatment (**A and B**) and cucumber planting (**C and D**). The bacterial and fungal phyla whose relative abundance was lower than 1% in all the soils were copolymerized in the “other” category. The asterisks denote significant differences between treatments (*P < 0.05, **P < 0.01), as determined by Duncan’s test.

As shown in Fig. 3, Proteobacteria, Firmicutes, Actinobacteria, Chloroflexi, and Gemmatimonadetes were the main bacterial phyla in all the soil samples and accounted for 72.95%–86.31% of the total bacterial community. Ascomycota, Basidiomycota, Mortierellomycota, and Chytridiomycota were the main fungal phyla in all the soil samples and accounted for 90.82%–97.78% of the total fungal community. Compared with that in the CK treatment, the relative abundance of Firmicutes significantly increased (*P* < 0.01) by 44.42%, 29.88%, and 48.67%, but the relative abundance of Actinobacteria significantly decreased (*P* < 0.01) by 53.64%, 50.89%, and 59.82% in the TS, BS, and PS treatments, respectively. In terms of fungal phyla, compared with that in the CK treatment, the relative abundance of Ascomycota in the TS, BS, and PS treatments significantly decreased (*P* < 0.01) by 8.97%, 10.04%, and 13.39%, respectively, but the relative abundance of Basidiomycota significantly increased (*P* < 0.01) by 75.51%, 108.04%, and 132.92%, respectively. After cucumber planting, the differences in the relative abundance of certain phyla, such as Firmicutes, Actinobacteria, Ascomycota, and Basidiomycota, among the treatments were similar to those after soil treatment.

#### Analysis of microbial community composition at the genus level

**Fig 4:**
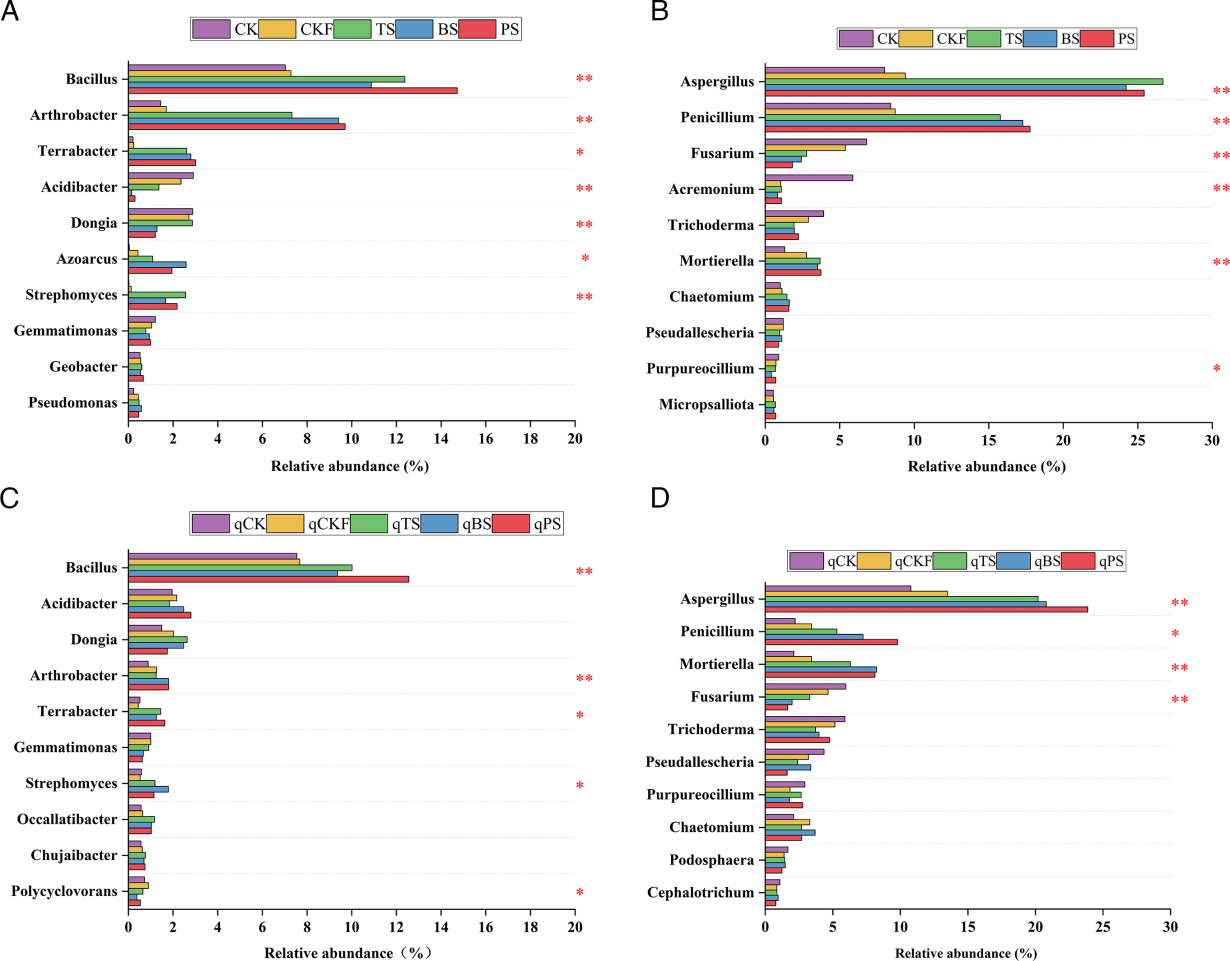
Relative abundance (%) of the top 10 main bacterial and fungal genera in the soils under different treatments after soil treatment (**A and B**) and cucumber planting (**C and D**). Asterisks denote significant differences between treatments (*P < 0.05, **P < 0.01), as determined by Duncan’s test.

As shown in Fig. 4, at the bacterial genus level, compared with the CK treatment, the TS, BS, and PS treatments significantly increased the relative abundance of *Bacillus*, *Arthrobacter*, *Terrabacter*, and *Streptomyces* but significantly reduced the relative abundance of *Acidibacter*. The relative abundance of *Bacillus* was greatest in the PS treatment group but exhibited no significant difference compared with those in the TS and BS groups. At the fungal genus level, compared with those in the CK treatment, the TS, BS, and PS treatments significantly increased the relative abundance levels of *Aspergillus*, *Penicillium*, and *Mortierella* but significantly reduced the relative abundance of *Fusarium*. The relative abundance of *Fusarium* was lowest in the PS treatment group but exhibited no significant difference compared with those in the TS and BS groups. After cucumber planting, the differences in the relative abundance of some genera, such as *Bacillus*, *Terrabacter*, *Mortierella*, and *Fusarium*, among the treatments were similar to those after soil treatment.

### Cooccurrence network analysis of microbial communities

**Fig 5:**
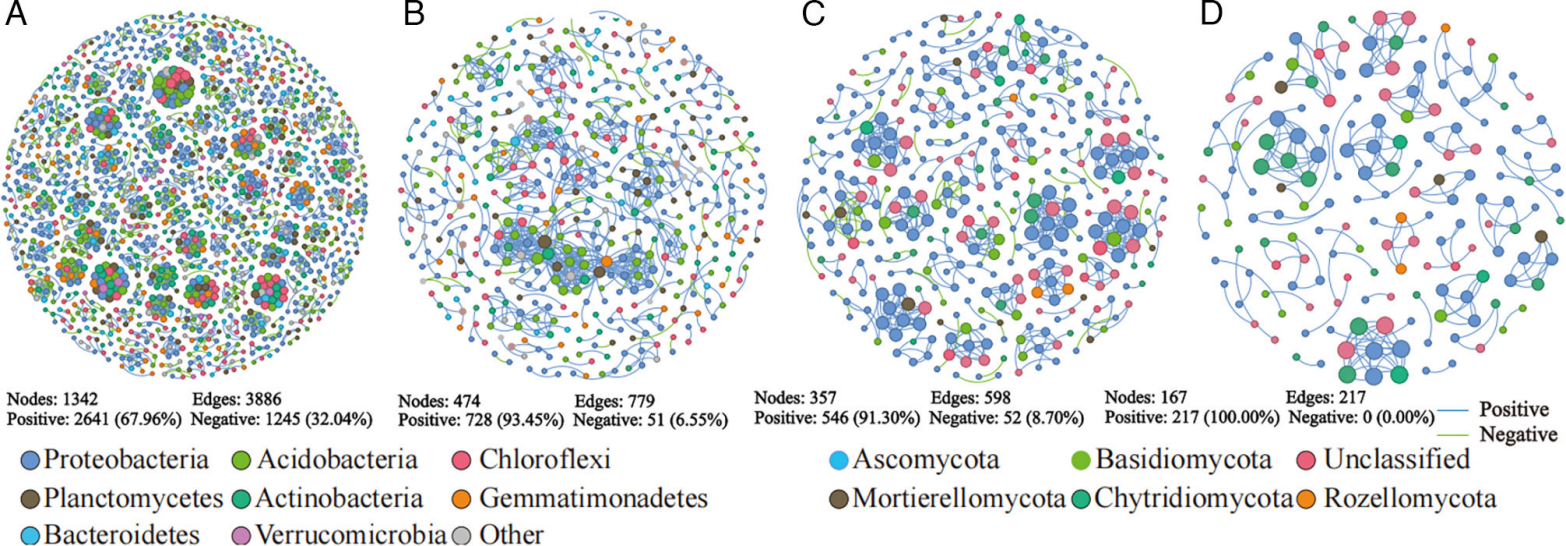
Cooccurrence networks of soil bacterial and fungal communities under different treatments. (**A**) Bacterial control group (qCK + qCKF). (**B**) Bacterial treatment group (qTS + qBS + qPS). (**C**) Fungal control group (qCK + qCKF). (**D**) Fungal treatment group (qTS + qBS + qPS). Each node represents an OTU; the node size is proportional to the relative abundance of the OTU; and node colors represent different fungal and bacterial phyla. The blue edges indicate positive correlations, while the yellow edges denote negative correlations.

On the basis of Spearman’s correlation analysis, cooccurrence networks of bacteria and fungi were established at the OTU level to reveal interactions between microorganisms. Notably, the number of nodes and edges in the bacterial networks was greater than that in the fungal networks. Compared with that in the control group (qCK + qCKF), the number of nodes and edges in the microbial network in the treatment group (qTS + qBS + qPS) decreased, but the number of positive interactions increased (Fig. 5).

### Prediction of soil microbial functions

**Fig 6:**
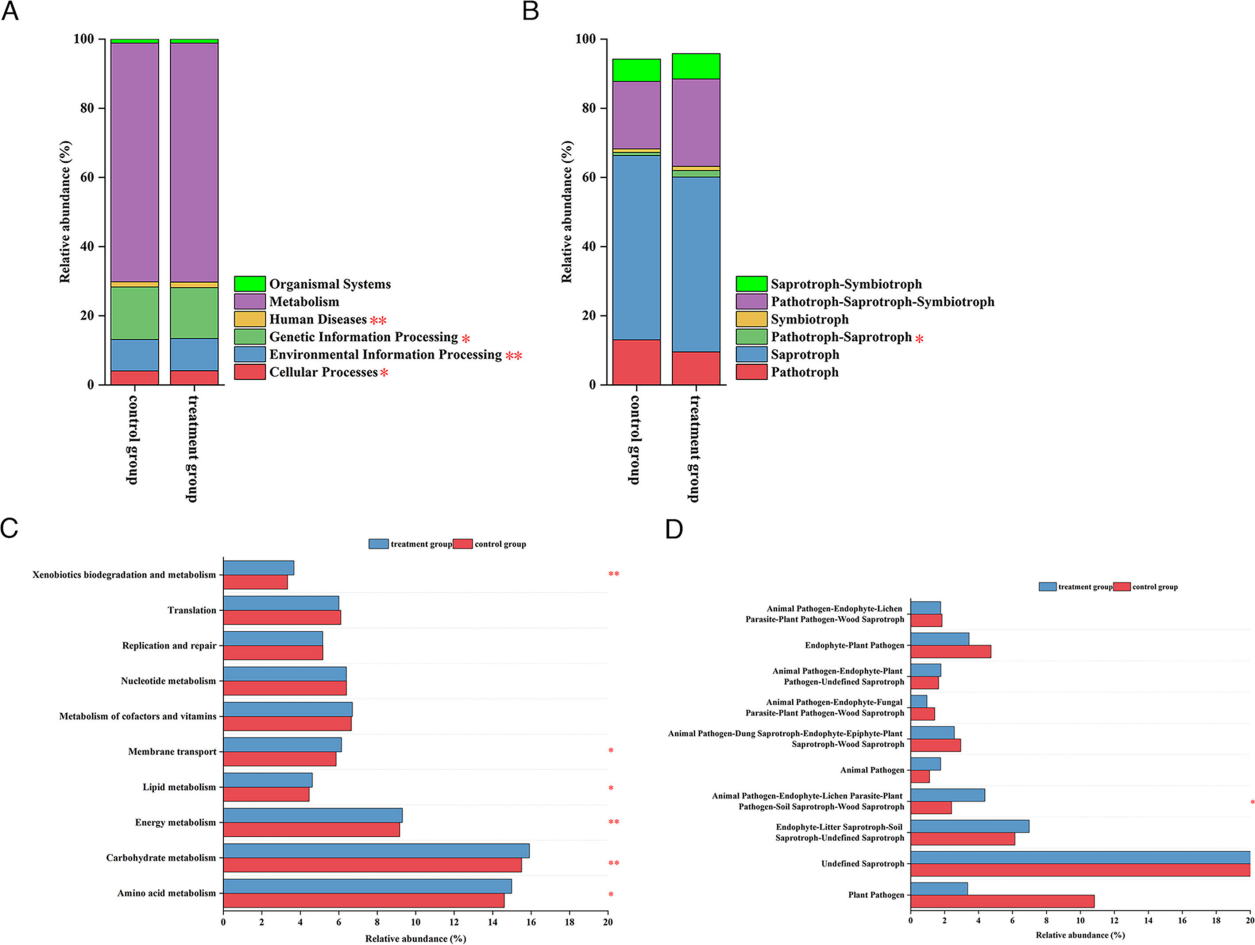
Prediction of soil bacterial and fungal functions under different treatments. (**A**) Bacterial control group (qCK + qCKF). (**B**) Bacterial treatment group (qTS + qBS + qPS). (**C**) Fungal control group (qCK + qCKF). (**D**) Fungal treatment group (qTS + qBS + qPS). Asterisks denote significant differences between treatments (*P < 0.05, **P < 0.01), as determined by the *t*-test.

With respect to the prediction of bacterial community function after cucumber cultivation using PICRUSt2 software combined with the KEGG database, six categories of level 1 functions were identified (Fig. 6A); compared with those in the control group (qCK + qCKF), the relative abundance levels of cellular processes, environmental information processing, genetic information processing, and human disease functions significantly increased in the treatment group (qTS + qBS + qPS). Forty-two categories of level 2 functions were identified, and we analyzed the differences in the metabolic functions of the top 10 relative abundances (Fig. 6C). Compared with those in the control group, the relative abundance levels of amino acid metabolism, carbohydrate metabolism, energy metabolism, lipid metabolism, membrane transport, and xenobiotic biodegradation and metabolism significantly increased in the treatment group.

With respect to the prediction of fungal community function after cucumber cultivation, the FUNGuild database revealed six categories of fungal functions (Fig. 6B). Pathotroph–saprotrophs were significantly more abundant in the treatment group than in the control group. Further analysis revealed that compared with the control group, the treatment group exhibited significantly greater numbers of animal pathogen–endophyte–lichen parasite–plant pathogen–soil saprotroph–wood saprotroph (Fig. 6D).

### Effects of different treatments on the yield and growth of cucumber

**Fig 7:**
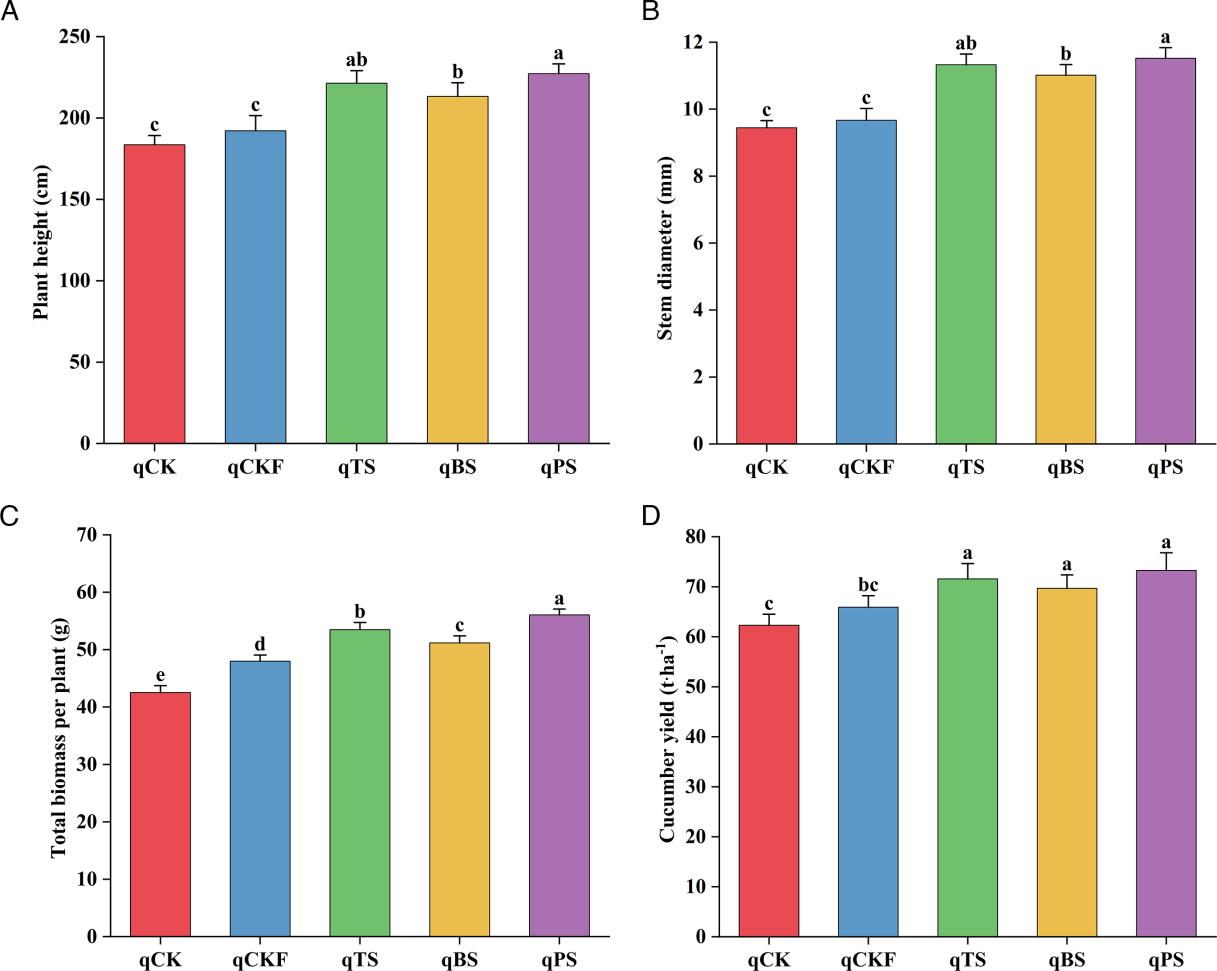
Plant height (**A**), stem diameter (**B**), total biomass per plant (**C**), and cucumber yield (**D**) under different treatments after cucumber planting. The different letters in the same figure indicate significant differences at *P* < 0.05, as determined by Duncan’s test. The error bars indicate the standard errors of the means of three replicates.

As shown in Fig. 7, the qTS, qBS, and qPS treatments significantly increased the cucumber plant height, stem diameter, total biomass per plant, and yield, with the qBS treatment yielding the greatest values. Compared with those in the qCK treatment, the cucumber plant height significantly increased (*P* < 0.05) by 20.59%, 16.12%, and 23.75%; the cucumber stem diameter significantly increased (*P* < 0.05) by 19.92%, 16.62%, and 21.98%; the cucumber total biomass per plant significantly increased (*P* < 0.05) by 25.79%, 20.34%, and 31.84%; and the cucumber yield significantly increased (*P* < 0.05) by 14.90%, 11.85%, and 17.59% in the qTS, qBS, and qPS treatments, respectively.

### Correlation between cucumber yield, soil chemical properties, soil enzyme activities, and microbial diversity

**Fig 8:**
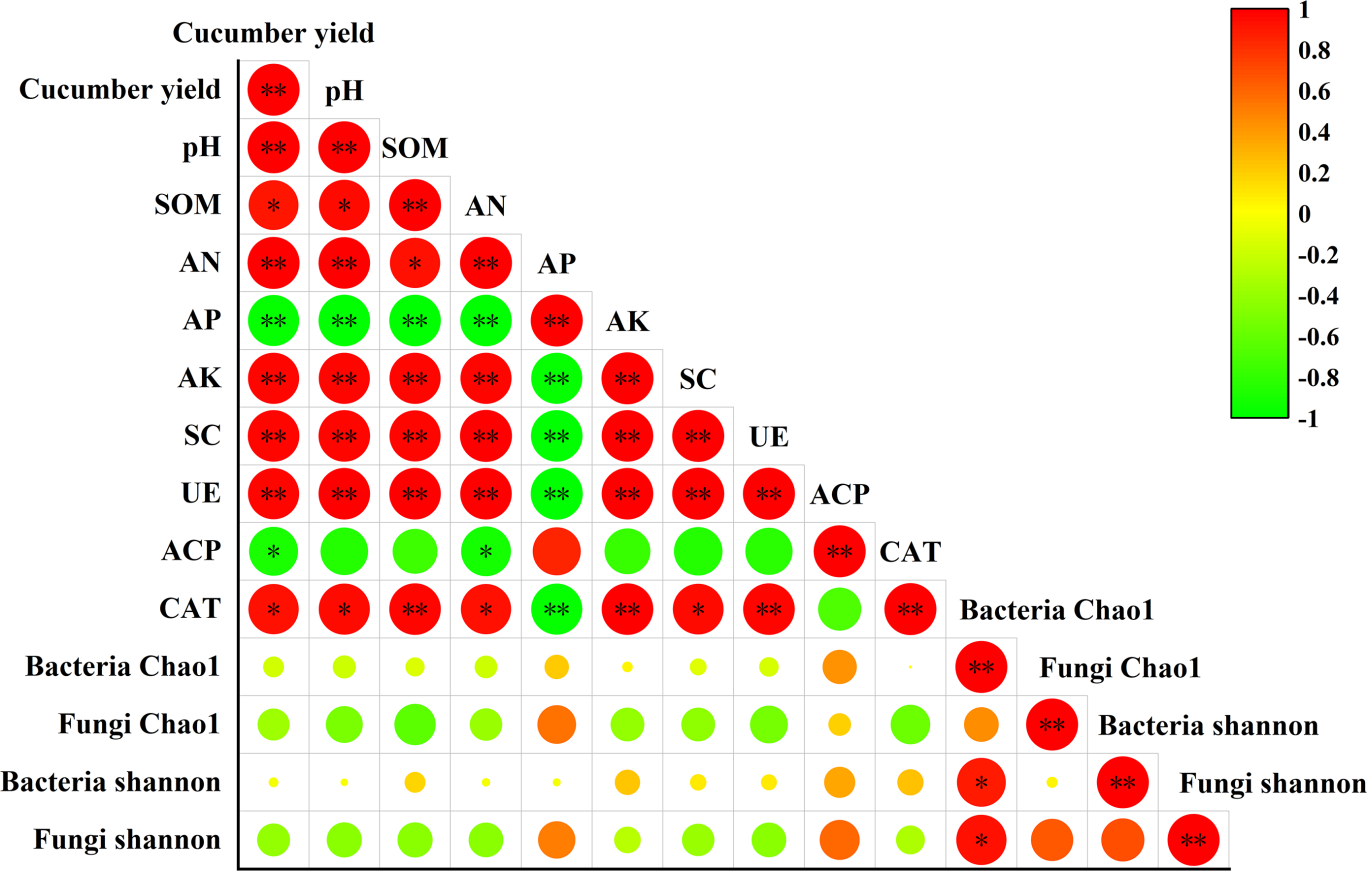
Relationships among cucumber yield, soil properties, soil enzyme activity, and microbial diversity. The figure shows Pearson’s correlation coefficients; the significance levels are as follows: *P < 0.05, **P < 0.01; blank represents a nonsignificant (*P* > 0.05) correlation.

The results of Pearson’s correlation analysis (Fig. 8) revealed that the soil pH; SOM, AN, and AK levels and the SC, UE, and CAT activities were significantly positively correlated with cucumber yield, whereas the soil AP content and ACP activity were significantly negatively correlated with cucumber yield. Furthermore, the soil pH was significantly positively correlated with the SOM, AN, and AK contents and SC, UE, and CAT activities. The soil AP content was significantly negatively correlated with the soil pH; SOM, AN, and AK levels; and SC, UE, and CAT activities. Moreover, the bacterial Chao1 index was significantly positively correlated with the bacterial Shannon index and the fungal Shannon index.

### Correlation between cucumber yield, soil chemical properties, soil enzyme activities, and microbial communities

**Fig 9:**
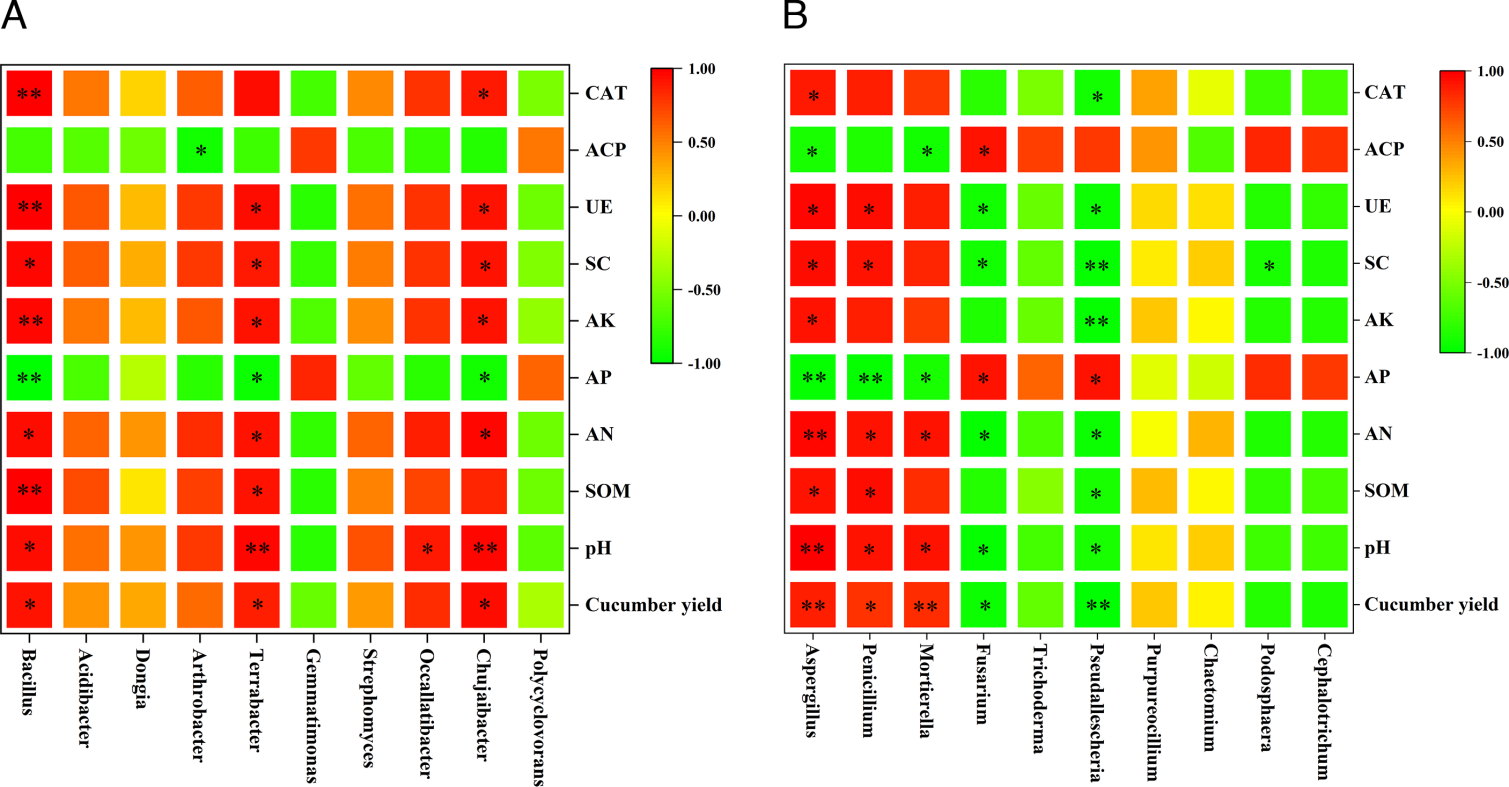
Correlation analysis between the main bacterial (**A**) and fungal (**B**) genera in the soil and cucumber yield, soil properties, and soil enzyme activities after cucumber planting. The significance levels are as follows: *P < 0.05, **P < 0.01; blank represents a nonsignificant (*P* > 0.05) correlation.

Pearson’s correlation analysis of the top 10 bacteria and fungi in terms of the relative abundance at the genus level with soil environmental factors and the cucumber yield indicated that *Bacillus* was significantly positively correlated with the cucumber yield; pH; SOM, AN, and AK levels; and SC, UE, and CAT activities and significantly negatively correlated with the AP content (Fig. 9A). *Penicillium* was significantly positively correlated with the cucumber yield; pH; SOM and AN levels; and SC and UE activities and significantly negatively correlated with the AP content. *Fusarium* was significantly positively correlated with the AP content and ACP activity and significantly negatively correlated with the cucumber yield, pH, AN content, and SC and UE activities (Fig. 9B).

## DISCUSSION

### Influences of RSD treatments on the soil chemical properties of cucumber

Soil chemical properties are among the main factors contributing to the occurrence of continuous cropping obstacles (38). As the number of consecutive years of cucumber cultivation increases, the soil pH decreases, and yields decrease accordingly (39). The soil pH increased significantly after RSD treatment because of H^+^ consumption via denitrification and other redox reactions under anaerobic conditions (40). In addition, the RSD treatment accelerated the turnover rate of inorganic nitrogen in the soil, resulting in increased soil nitrogen and other inorganic nutrient levels (41). Moreover, studies have shown that the soil pH is negatively correlated with the soil available phosphorus content and that the increase in soil pH after RSD treatment may explain the decrease in soil available phosphorus. Furthermore, the decrease in soil acid phosphatase activity may also explain the decrease in soil available phosphorus (14, 42).

### Effects of RSD treatments on the soil enzyme activities of cucumber

Soil enzymes are the most biologically active substances in ecological processes such as ecosystem material cycling and energy flow and are important promoters of soil metabolism, which, together with microorganisms, promote the metabolic processes of soil (43). In soil with crop continuous cropping obstacles, the activities of soil UE, SC, and CAT decrease (44). Our research revealed that RSD treatment increased the activities of soil UE, SC, and CAT, which is consistent with the results of Zhan et al. (45). Studies have shown that soil SC and UE activities are significantly positively correlated with the contents of AN, SOM, and AK in the soil (46). Our study revealed that soil SC, UE, and CAT activities were significantly positively correlated with soil pH as well as SOM, AN, and AK contents.

### Impact of RSD treatments on the soil microbial communities of cucumber

Soil microbial communities are essential for nutrient cycling, plant growth, and disease prevention and control (47). Our results revealed that RSD treatments reduced microbial diversity and richness, possibly because the anaerobic environment created by RSD treatment inhibited the growth of aerobic microorganisms (33). In addition, cooccurrence network analysis revealed that the number of nodes decreased with increasing RSD. Although RSD treatment reduces microbial community diversity and abundance, this treatment also cultivates a unique core microbiota (18). The relative abundance levels of *Bacillus*, *Arthrobacter*, and *Streptomyces* increased in the RSD treatment group. Members of the genera *Bacillus* and *Arthrobacter* inhibit the growth of pathogenic microorganisms by producing lipopeptides and bacteriocins (48, 49). Furthermore, *Streptomyces* has been found to successfully control *Fusarium oxysporum*- and *Botrytis cinerea*-induced soil-borne diseases (50). Ascomycota was dominant in the soil of greenhouses or plastic sheds exposed to continuous cropping (51, 52), and our findings corroborated that Ascomycota dominated in these treatments. According to numerous studies, RSD treatments can effectively decrease soil-borne pathogens (53, 54), which is consistent with our findings that *Fusarium* abundance significantly decreased in the RSD treatments. The relative abundance of *Aspergillus*, *Penicillium*, and *Mortierella* increased in the RSD treatments. Some studies have shown that *Penicillium* can degrade harmful substances present in soil, and its secondary metabolites have been demonstrated to significantly inhibit certain pathogens (55).

### Effects of RSD treatments on the growth, development, and yield of cucumber

The C/N ratio is a key factor influencing the effectiveness of RSD treatment (56). Tan et al. (21) used alfalfa (C/N ratio: 14.47), maize straw (C/N ratio: 33.80), and rice straw (C/N ratio: 68.61) as organic materials for RSD treatment and reported that maize straw was the most effective organic material. A low C/N of organic matter can cause ammonia toxicity. A high C/N ratio of organic matter may lead to nitrogen loss, and the optimal substrate C/N ratio may be approximately 30 (21, 57). Our research yielded similar results; notably, among the three types of vegetable straw, pepper straw (C/N ratio: 27.85) was the most effective. In this study, the qTS, qBS, and qPS treatments promoted the growth, development, and yield of cucumber. This may be related to the fact that the RSD treatment improved the soil properties and altered the structure of the soil microbial community (58). Beneficial microorganisms inhibit pathogens and reduce cucumber diseases, which are also important factors in promoting cucumber growth and development (40, 41). Furthermore, we used readily available vegetable straw as the source of organic material for our RSD treatments, effectively converting waste into a valuable resource for the RSD process and offering a promising avenue for the efficient use of vegetable straw (59).

### Conclusion

A year of field trials revealed that the use of vegetable straw as organic material for RSD treatment can effectively alleviate greenhouse cucumber continuous cropping obstacles. The RSD treatment increased the soil pH; the SOM, AN, and AK contents; and SC, UE, and CAT activities. In addition, RSD treatment altered the soil microbial community structure and increased the relative abundance of *Bacillus*, *Arthrobacter*, *Streptomyces*, and *Penicillium*, whereas the relative abundance of *Fusarium* decreased. RSD treatment promoted the growth and development of cucumber and increased the yield, and pepper straw was the most effective organic material for RSD treatment. However, the long-term effects of RSD treatments on soil properties and microbial communities must be studied.

## Data Availability

The original data have been deposited in the NCBI database with the accession numbers PRJNA1164675 and PRJNA1164809 for the 16S rRNA and ITS genes, respectively.
